# Epidemiology of Dengue among Children Aged < 18 Months—Puerto Rico, 1999–2011

**DOI:** 10.4269/ajtmh.15-0382

**Published:** 2016-02-03

**Authors:** Anne M. Hause, Janice Perez-Padilla, Kalanthe Horiuchi, George S. Han, Elizabeth Hunsperger, Jonathan Aiwazian, Harold S. Margolis, Kay M. Tomashek

**Affiliations:** Dengue Branch, Division of Vector-Borne Diseases, Centers for Disease Control and Prevention, San Juan, Puerto Rico

## Abstract

Dengue, a mosquito-borne viral illness caused by dengue virus types (DENV)-1 to DENV-4, is endemic in Puerto Rico. Severe dengue usually occurs in individuals previously infected with DENV or among infants born to previously infected mothers. To describe clinical features of dengue in infants, we retrospectively characterized dengue patients aged < 18 months reported to the Passive Dengue Surveillance System (PDSS) during 1999–2011. To determine frequency of signs, symptoms, and disease severity, case report forms and medical records were evaluated for patients who tested positive for dengue by reverse transcriptase polymerase chain reaction or anti-DENV immunoglobulin Menzyme-linked immunosorbent assay. Of 4,178 reported patients aged < 18 months, 813 (19%) were laboratory positive. Of these, most had fever (92%), rash (53%), bleeding manifestations (52%), and thrombocytopenia (52%). Medical records were available for 145 (31%) of 472 hospitalized patients, of which 40% had dengue, 23% had dengue with warning signs, and 33% had severe dengue. Mean age of patients with severe dengue was 8 months. Anti-DENV immunoglobulin G (IgG) titers were not statistically different in patients with (50%) and without (59%) severe dengue. In this study, one-third of DENV-infected infants met the severe dengue case definition. The role of maternal anti-DENV IgG in development of severe disease warrants further study in prospective cohorts of mother-infant pairs.

## Introduction

Dengue is a mosquito-borne disease caused by one of four closely related dengue virus serotypes (DENV-1 through -4).^1^ Infection with any DENV can lead to inapparent infection, undifferentiated acute febrile illness (AFI), dengue, or severe dengue, including dengue hemorrhagic fever (DHF) and dengue shock syndrome (DSS).^2^ Severe dengue most often occurs among patients who have been previously infected with a DENV (i.e., secondary DENV infection).^2^ Children aged < 1 year (i.e., infants) with primary DENV infection who are born to mothers who have been previously infected with a DENV also experience high rates of severe dengue.^3^ The most common explanation for this finding is antibody-dependent enhancement of disease (ADE), in which subneutralizing/non-neutralizing levels of anti-DENV immunoglobulin G (IgG) antibodies acquired from a previous DENV infection (or, in the case of infants, from their mother in utero) bind to a different DENV serotype but do not neutralize it. This then results in enhanced viral uptake and infection in Fc receptor-bearing cells, specifically mononuclear phagocytes.^1^,^3^–^10^ This is thought to enable an increase in virus replication resulting in increase in viral load, which triggers a host inflammatory response leading to severe disease manifestations including shock due to plasma leakage and bleeding.^1^,^4^,^5^,^8^,^11^

Infants are a vulnerable population in areas with endemic dengue. They are at increased risk for DHF compared witholder children, with incidence peaking around age 6–8 months.^3^,^8^,^11^,^12^ Compared with older children with DHF, infants are more likely to develop medical complications, require longer hospital stays, and succumb to the illness.^11^,^13^ However, unlike patients with DHF who are experiencing secondary DENV infection, infants with dengue do not have virus-specific memory B- and T-cells, and thus maternally derived anti-DENV IgG serves as the primary protection against DENV infection. Because the incidence of DHF and in vitro replication of DENV in mononuclear phagocytes obtained from ill infants both peak at age 6–8 months,^3^ it has been hypothesized that ADE plays a central role in the pathogenesis of severe dengue in infants.^3^

The relationship between anti-DENV IgG subclass and dengue severity has been examined.^14^ Of the four subclasses, high levels of anti-DENV IgG1 have been associated with severe disease in adults. This IgG subclass plays an important role in complement activation and, consequently, in the cytokine cascade. Because IgG1 is the subclass most efficiently transported between mother and fetus via the placenta and infants have a greater proportion of anti-DENV IgG1, IgG1 may therefore participate in the mechanism of severe dengue in infants.^14^–^16^

In Puerto Rico, infants typically have one of the highest age-group specific incidences of dengue.^17^ In this report, we describe the epidemiology of dengue among children aged < 18 months in Puerto Rico who were reported to the island-wide Passive Dengue Surveillance System (PDSS) and had illness onset during January 1, 1999–December 31, 2011. We sought to determine whether anti-DENV IgG titers and subclass during the acute phase of the disease were associated with disease severity among hospitalized patients.

## Methods

Study data were obtained from PDSS, which has been jointly operated by the Centers for Disease Control and Prevention, Dengue Branch (CDC-DB) and Puerto Rico Department of Health for > 40 years.^18^ All suspected dengue patients reported to PDSS aged < 18 months and with symptom onset during January 1, 1999–December 31, 2011 were included in the study. For patients reported to PDSS, data including demographic information, onset date, signs and symptoms, clinical outcomes, and clinical laboratory data are collected via the dengue case investigation form (DCIF) (www.cdc.gov/dengue/resources/dengueCaseReports/DCIF_English.pdf). Serum specimens are submitted along with the DCIF to enable diagnostic testing. Specimens collected ≤ 4 days after disease onset were tested by DENV type-specific, real-time reverse transcriptase polymerase chain reaction (rRT-PCR), and specimens collected ≥ 6 days after disease onset were tested by anti-DENV immunoglobulin M (IgM) capture enzyme-linked immunosorbent assay (MAC-ELISA).^19^,^20^ Specimens collected 5 days after disease onset were tested by both rRT-PCR and MAC-ELISA.

All specimens used in the study were retested using the above-mentioned algorithm to account for the historical differences in testing algorithms that had been used over time. If specimen volume permitted, anti-DENV IgG titration and subclass testing was performed. Relative amount of anti-DENV IgG in specimen was measured using anti-DENV IgG titration ELISA.^21^ A positive anti-DENV IgG titer was defined by a reciprocal titer > 40. The four human anti-DENV IgG subclasses were identified by immunoassay (PeliClass Human IgG Subclass kit, Sanquin, Amsterdam, the Netherlands).

To more accurately classify patients using World Health Organization (WHO, 2009) case definitions,^2^,^22^ medical records were reviewed from all hospitalized, laboratory-positive dengue patients aged < 18 months who were reported to PDSS during the first 5 days after illness onset. Cases presenting during the first 5 days after illness onset were selected for review so that remaining specimen could be tested for the presence of anti-DENV IgG that was likely to be maternal in origin. Medical records were abstracted using a standardized form that collected data including birth history, medical history since birth, clinical course, and clinical signs and symptoms.

### Definitions.

A *suspected dengue case* is a dengue-like AFI affecting a person for whom a health-care provider suspected dengue and submitted a DCIF and serum specimen to PDSS.

A *laboratory-positive case* is a suspected dengue case for which DENV nucleic acid was detected by rRT-PCR or anti-DENV IgM was detected by MAC-ELISA.

A *laboratory-negative case* is a suspected case with no anti-DENV IgM detected by MAC-ELISA in a specimen collected ≥ 6 days after illness onset.

A *laboratory-indeterminate case* is a suspected case for which DENV nucleic acid was not detected in a specimen collected in the first 5 days after illness onset and a specimen ≥ 6 days after illness onset was not submitted.

*Severe dengue, dengue*, and *warning signs of severe dengue* were defined according to 2009 WHO guidelines, and dengue fever (DF), DHF, and DSS were defined according to 1997 WHO guidelines.^2^,^22^

*Bleeding manifestations* was defined as having epistaxis, mucosal bleeding, gum bleeding, hematuria, hematemesis, melena, hematochezia, or menorrhagia.

### Statistical analysis.

The frequencies of clinical, demographic, and laboratory data were calculated for laboratory-positive and laboratory-negative cases. Univariate and multivariate analyses were performed to assess possible associations between positive or negative laboratory status and symptoms of severe dengue. Continuous variables were compared using Student's *t* test, the Mann–Whitney *U* test, and linear regression. Categorical variables were evaluated using Fisher's exact test. Comparison of the information collected on the DCIF and from the hospital chart of the same patient was done using the paired Student's *t* test of means and McNemar's test for comparison of proportions. Relative risk was calculated using the RelativeRisk package in R. The kappa statistic was used as a measure of agreement between dengue definitions. *P* values ≤ 0.05 were considered statistically significant, and all confidence intervals (CIs) were calculated at the 95% level. Data were analyzed using STATA (STATA^®^ Corporation, College Station, TX), SAS^®^ version 9.2 (SAS Institute, Cary, NC), and R version 3.0.1 (R Foundation for Statistical Computing, Vienna, Austria).

### Ethical review.

The study protocol was reviewed and approved by the CDC's Human Subjects Institutional Review Board (IRB 6295, May 9, 2012).

## Results

**Figure 1.**
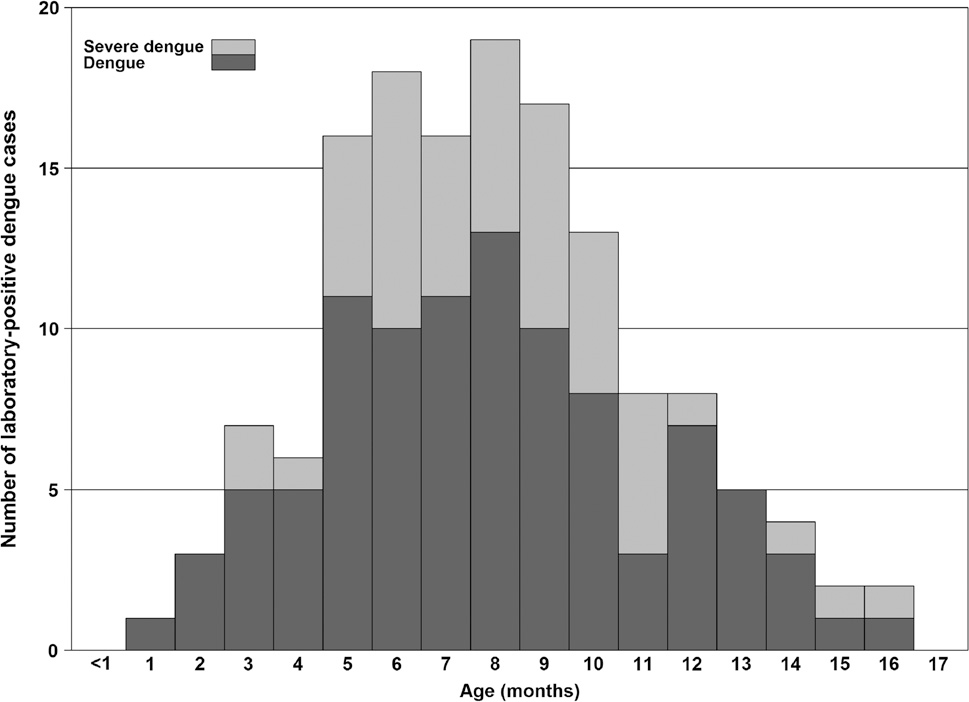
Laboratory-positive dengue cases by disease severity (both World Health Organization 1997 and 2009 case definition) based on data reported to the passive dengue surveillance system in Puerto Rico, 1999–2011.

Of 4,178 suspected dengue cases reported during January 1, 1999–December 31, 2011, a total of 813 (20%) were laboratory positive, 737 (18%) were laboratory negative, 2,475 (59%) were laboratory indeterminate, and 153 (4%) were unable to be tested. A slightly larger proportion of laboratory-positive cases were male (58%) (Table 1). Patients who were identified as a laboratory-positive case were significantly younger than those who were identified as laboratory-negative cases (8.6 versus 10.6 months, respectively). The age distribution of laboratory-positive cases was approximately symmetric with a peak at 8 months (Figure 1

). Conversely, the age distribution of laboratory-negative cases was nonsymmetrical.

Clinical signs and symptoms indicative of dengue were more commonly reported among laboratory-positive cases than laboratory-negative cases (Table 1). Most laboratory-positive cases had fever (92%), rash (53%), bleeding manifestations (52%), and thrombocytopenia (52%). Laboratory-positive cases were two times more likely than laboratory-negative cases to have petechiae, ecchymosis, any bleeding manifestation, thrombocytopenia, or plasma leakage. Of interest, laboratory-positive cases were also more likely than laboratory-negative cases to have vomiting and diarrhea. Cough was similarly common in laboratory-positive and laboratory-negative (44% versus 48%, respectively) cases. Among the laboratory-positive cases, there were seven fatal outcomes, compared with two fatal outcomes among the laboratory-negative cases.

Of 472 (58%) laboratory-positive cases who were reported to have been hospitalized, 145 (31%) had medical records that were available and reviewed (Table 2). Of the 145 cases who were reviewed, 41% met the 1997 WHO case definitions for DF, 19% for DHF, and 4% for DSS. When using the 2009 WHO case definitions, 40% met the case definitions for dengue, 23% for dengue with warning signs, and 33% for severe dengue. When compared with the data from the chart review, the percentage of underreporting for each of these respective classes of severe dengue based on data reported through PDSS was 3% for DSS, 15% for DHF, and 21% for severe dengue.

The most common warning signs of dengue among these 145 cases were pleural effusion (25%), persistent vomiting (18%), and ascites (14%). Of those infants with severe dengue, 3 (6%) had severe bleeding, 25 (52%) had severe plasma leakage resulting in shock or respiratory distress, 5 (10%) had severe organ impairment, and 15 (31%) met the definitions for two or more of these categories. Other clinical signs were refusal to eat (50%), irritability (41%), constant crying (15%), and changes in sleeping habits (8%). Severe dengue and DHF/DSS cases presented to the hospital and were hospitalized 3.0 (95% CI = 2.5–3.6) and 2.6 (95% CI = 1.9–3.3) days postsymptom onset, respectively; their length of hospitalization was similar at 7.9 (95% CI = 6.4–9.4) and 6.9 (95% CI = 5.7–8.1) days, respectively.

Cases with DHF were not more likely (relative risk ratio [RR] = 1.1; 95% CI = 0.3–3.0) to have a positive anti-DENV IgG titer than those not meeting criteria for DHF. Similarly, cases with severe dengue were not more likely (RR = 1.1; 95% CI = 0.4–2.1) to have detectable anti-DENV IgG antibody than those not meeting criteria for severe dengue. Anti-DENV IgG1 was found to be the most abundant subclass identified, followed by IgG2 (Supplemental Tables 1 and 2). Subclass of anti-DENV IgG was not significantly associated with any classification of disease severity (data not shown).

## Discussion

In examining the epidemiology of dengue among children aged < 18 months in Puerto Rico, we found that laboratory-positive dengue cases were significantly younger than laboratory-negative cases. We identified several clinical features that were more common among hospitalized laboratory-positive cases than laboratory-negative cases. These features, which were largely objective signs, may prove to be useful clinical indicators of dengue in this preverbal age group, as the majority of cases failed to meet case criteria for dengue. We found that children in our population typically acquired DHF slightly later than children in other areas with endemic dengue. Additionally, we found that severe dengue in this age group was underestimated through passive surveillance.

Most studies have observed an association of severe dengue with infants aged 6–8 months, when maternal IgG wanes and ADE occurs.^3^,^8^,^11^,^12^,^23^–^25^ Similarly, the mean age of DHF and severe dengue among our population was 7.7 months and 8.6 months, respectively, and most severe dengue cases occurred in children aged 6–10 months. The slightly wider age range of severe dengue cases may be explained by a lower force of infection in Puerto Rico than in areas with highly endemic dengue. Consequently, anti-DENV IgG titers may remain high into women's childbearing years, resulting in their infants being exposed to higher titers of anti-DENV IgG in utero that are then catabolized in the infant over a longer period of time. This could result in severe dengue occurring slightly later in these infants.

We also observed that young children hospitalized with DHF or severe dengue had similar rates of detection of anti-DENV IgG antibody. Consistent with the subclass distribution of IgG antibody passively transferred from mother to fetus via placenta, anti-DENV IgG1 and IgG2 were the most abundant subclasses identified in our study population.^15^,^16^ Moreover, evaluating anti-DENV IgG subclass by disease severity yielded no statistically significant associations.

A strength of this study was that we had a large number of laboratory-positive cases, and the medical record review allowed for a more complete description of patients' clinical course and consequently more accurate classification by case definition. However, much of our retrospective study relied on existing clinical data collected via passive dengue surveillance, which can be incomplete and underestimate ultimate severity. In addition, a limitation of this study was that anti-DENV IgG antibody titer and subclass testing could not be performed for half of the patients due to insufficient specimen volume, which is a common obstacle for this age group.^9^ The resulting sample size limited our ability to effectively evaluate anti-DENV IgG antibody titer and subclass distribution. In addition, because our subanalysis included only patients who were hospitalized, some selection bias may exist with regard to the incidence of severe dengue. However, if we were able to review medical records from all children aged < 18 months, regardless of admission status we may expect to find an even higher rate of severe dengue among this cohort of young children.

We have described clinical signs and symptoms among children aged < 18 months suspected to have dengue and reported to PDSS. In comparing the signs and symptoms reported to PDSS and those documented in the medical record, we found that PDSS underestimates severity among hospitalized laboratory-positive dengue cases. Among hospitalized laboratory-positive cases, we did not observe an association between the presence of detectable anti-DENV IgG or IgG subclass and the occurrence of DHF and severe dengue. To determine the true association between maternal anti-DENV antibody decay and onset of severe dengue in infants, prospective studies should be conducted that follow pregnant mothers through to birth.

## Supplementary Material

Supplemental Tables.

## Figures and Tables

**Table 1:** Demographic and clinical features of laboratory-positive and laboratory-negative dengue cases aged < 18 months reported to the Passive Dengue Surveillance System, Puerto Rico, 1999–2011

	Dengue laboratory positive		Dengue laboratory negative		Difference (95% CI)
	*N* = 813		*N* = 737		
Mean age at onset in months (95% CI)	8.6 (8.4–8.9)		10.6 (10.2–10.9)		−1.9 (−2.3 to 1.5)*
	*N*	(%)	*N*	(%)	Relative risk (95% CI)
Male gender	468	58	412	56	1.0 (0.9–1.1)
Fatal outcome	7	1	2	1	1.4 (1.0–2.1)
Fever	750	92	564	77	3.2 (2.3–4.4)†
Rash	428	53	167	23	2.0 (1.8–2.2)†
Petechiae	384	47	89	12	2.3 (2.0–2.5)†
Ecchymosis	69	8	6	1	1.9 (1.8–2.1)†
Bleeding manifestations‡	419	52	96	13	2.3 (2.1–2.5)†
Thrombocytopenia	424	52	76	10	3.1 (2.7–3.6)†
Plasma leakage	105	13	22	3	1.7 (1.5–1.8)†
Shock	49	6	13	2	1.5 (1.3–1.8)†
Jaundice	17	2	5	1	1.5 (1.2–1.9)†
Vomiting	312	38	195	26	1.3 (1.2–1.5)†
Diarrhea	304	37	201	27	1.3 (1.2–1.4)†
Nasal congestion	302	37	359	49	0.8 (0.7–0.9)†
Cough	355	44	357	48	1.0 (0.9–1.1)

CI = confidence interval.

* Welch's two-sample t test.

† *P* ≤ 0.05.

‡ Epistaxis, mucosal bleeding, gum bleeding, hematuria, hematemesis, melena, hematochezia, or menorrhagia.

**Table 2:** Comparison of dengue severity as reported in the surveillance case report vs. the medical chart for the 145 hospitalized, laboratory-positive children aged < 18 months who were reported to the Passive Dengue Surveillance System and whose charts were reviewed, Puerto Rico, 1999–2011

Diagnosis	Case report (%)	Chart review (%)	% Difference (95% CI)	*P* value*
DF†	74 (51)	59 (41)	10 (−5.7 to 26.4)	0.13
DHF†	6 (4)	27 (19)	−15 (−24.8 to 4.2)	< 0.01
DSS†	2 (1)	6 (4)	−3 (−8.5 to 3.0)	0.29
Dengue‡	75 (52)	58 (40)	12 (−4.3 to 27.7)	0.05
Dengue with warnings signs‡	10 (7)	34 (23)	−16 (−28.1 to 5.0)	< 0.01
Severe dengue‡	18 (12)	48 (33)	−21 (12.4 to 33.1)	< 0.01

CI = confidence interval; DF = dengue fever; DHF = dengue hemorrhagic fever; DSS = dengue shock syndrome; WHO = World Health Organization.

* *P* value calculated from McNemar's test for proportions.

† Defined according to WHO 1997 Guidelines.

‡ Defined according to WHO 2009 Guidelines.
