# Supplementary material for: Epidemiology of Dengue among Children Aged < 18 Months—Puerto Rico, 1999–2011
Source: Am J Trop Med Hyg. 2016 Feb 3;94(2):404–8. doi: 10.4269/ajtmh.15-0382 (PMC4751950; doi:10.4269/ajtmh.15-0382)
Supplement: Supplementary file 1 [file SD5.pdf]

Supplemental Table 1

Distribution of average anti-DENV IgG subclass titers* among 145 children aged < 18 months with DHF or DF (as determined by medical record review) in serum specimens collected 0–5 days postdisease onset, Puerto Rico, 1999–2011

Anti-DENV IgG	DHF† (*N* = 4)	DF or AFI (*N* = 5)	Difference of means (95% CI)
IgG1	2.17	1.73	0.44 (−4.86 to 5.74)
IgG2	0.67	0.03	0.64 (−2.98 to 4.26)
IgG3	0.18	0.08	0.10 (−0.25 to 0.44)
IgG4	0.03	0.04	−0.01 (−0.14 to 0.11)
	(*N* = 14)	(*N* = 19)	
Overall IgG titer	1.07	0.53	0.55 (−0.34 to 1.43)

AFI = acute febrile illness; CI = confidence interval; DENV = dengue virus serotype; DF = dengue fever; DHF = dengue hemorrhagic fever; IgG = immunoglobulin G; WHO = World Health Organization.
*$\log_{10}$ titers are shown.
†DHF comparison group includes all children with dengue fever or undifferentiated febrile illness as defined by the 1997 WHO case definitions.

Supplemental Table 2

Distribution of average $\log_{10}$ anti-DENV IgG subclass titers* among 145 children aged < 18 months with severe or nonsevere dengue (as determined by medical record review) in serum specimens collected 0–5 days postdisease onset, Puerto Rico, from 1999 to 2011

Anti-DENV IgG	Severe dengue† (*N* = 6)	Nonsevere dengue (*N* = 5)	Difference of means (95% CI)
IgG1	3.98	2.85	1.13 (−8.95 to 11.21)
IgG2	0.97	0.57	0.40 (−3.19 to 3.98)
IgG3	1.69	0.14	1.55 (−4.56 to 7.65)
IgG4	< 0.01	0.06	−0.06 (−0.17 to 0.05)
	(*N* = 24)	(*N* = 19)	
Overall IgG titer	0.92	0.47	0.44 (−0.22 to 1.11)

CI = confidence interval; DENV = dengue virus serotype; IgG = immunoglobulin G; WHO = World Health Organization.
*$\log_{10}$ titers are shown.
†Severe dengue comparison group includes all children with dengue or dengue with warning signs as defined by the 2009 WHO case definitions.
